# Fungi-based treatment of brewery wastewater—biomass production and nutrient reduction

**DOI:** 10.1007/s00253-017-8185-9

**Published:** 2017-02-17

**Authors:** M. Hultberg, H. Bodin

**Affiliations:** 1 Department of Biosystems and Technology, Swedish University of Agricultural Sciences, P.O. Box 103, SE 230 53 Alnarp, Sweden; 2 Division of Natural Sciences, Kristianstad University, Kristianstad, Sweden

**Keywords:** Filamentous fungi, Microbrewery, Nutrient recycling, *Pleurotus ostreatus*, *Trichoderma harzianum*, Water quality

## Abstract

The beer-brewing process produces high amounts of nutrient-rich wastewater, and the increasing number of microbreweries worldwide has created a need for innovative solutions to deal with this waste. In the present study, fungal biomass production and the removal of organic carbon, phosphorus and nitrogen from synthetic brewery wastewater were studied. Different filamentous fungi with a record of safe use were screened for growth, and *Trametes versicolor*, *Pleurotus ostreatus* and *Trichoderma harzianum* were selected for further work. The highest biomass production, 1.78 ± 0.31 g L^−1^ of dry weight, was observed when *P. ostreatus* was used for the treatment, while *T. harzianum* demonstrated the best capability for removing nutrients. The maximum reduction of chemical oxygen demand, 89% of the initial value, was observed with this species. In the removal of total nitrogen and phosphorus, no significant difference was observed between the species, while removal of ammonium varied between the strains. The maximum reduction of ammonium, 66.1% of the initial value, was also found in the *T. harzianum* treatment. It can be concluded that all treatments provided significant reductions in all water-quality parameters after 3 days of growth and that the utilisation of filamentous fungi to treat brewery wastewater, linked to a deliberate strategy to use the biomass produced, has future potential in a bio-based society.

## Introduction

Beer is the fifth most consumed beverage in the world, and the beer-brewing industry constitutes an important economic segment in many countries around the world (Fillaudeau et al. 2006; Simate et al. 2011). In recent years, the interest in small-scale brewing has also grown due to a rise in the appeal of locally produced food and beverages (Maier 2013), generally as a result of an increase in the environmental consciousness of individual consumers (Schnell and Reese 2003).

The brewing process consumes large quantities of water and generates 3–10 L of wastewater per 1 L of beer produced (Simate et al. 2011; Olajire 2012; Seluy and Isla 2014). This wastewater typically contains very high levels of organic carbon and phosphorus, and nitrogen levels similar to or higher than those found in raw domestic wastewater (Hang et al. 1975; Brewers of Europe 2002; Rao et al. 2007; Simate et al. 2011). However, compared to municipal sewage, brewery wastewater contains high-quality nutrients, but not problematic pollutants such as pharmaceuticals and enteric pathogens.

For microbreweries, wastewater volumes range from 18 to 3000 m^3^ per day, depending on the size of the microbrewery (Tucker 2007; Yang and Li 2002). Currently, microbreweries may discharge 90% of their wastewater directly into municipal sewer systems (Shao et al. 2008) or to the surrounding environment in countries with less developed wastewater treatment systems (Yang and Li 2002; Simate et al. 2011), often without prior on-site treatment. Discharge of brewery wastewater into municipal sewer systems can create odour and corrosion management issues and also increase greenhouse gas emissions from such systems (Sudarjanto et al. 2011). The discharge will also evidently increase the load of biodegradable organic carbon and nutrients to municipal wastewater treatment plants, and problems related to the discharge of brewery wastewater have been observed in several wastewater treatment plants in Sweden (IVL 2002). For large-scale breweries with existing wastewater treatment on-site, conventional treatment methods have been anaerobic reactors (UASB) and/or activated sludge systems (Zheng et al. 2015; Simate et al. 2011). These treatment systems generally do not lead to recycling of important nutrients in the waste, but instead produce relatively large quantities of low-value sludge and create disposal problems (Simate et al. 2011; Shao et al. 2008). Also, the use of microalgae for treatment of brewery wastewater has been investigated (Mata et al. 2012). However, the constraints are microalgal harvest (Uduman et al. 2010) and the risk for low transmittance of light due to particles in the brewery wastewater.

The nutritional composition of brewery wastewater suggests that it could be a suitable medium for cultivation of heterotrophic microorganisms such as fungi. In fact, submerged cultivation of fungi is a biotechnology that has been explored in the past, where liquid medium is inoculated with spores or mycelium from certain fungi. Simultaneous cultivation of fungal biomass and waste treatment, such as the high removal of organic carbon in brewery wastes, has also been reported using this technique (Shannon and Stevenson 1975; Hang et al. 1975). However, in the last three decades, submerged cultivation of fungal biomass has received less attention, despite the use of filamentous fungi for wastewater treatment being identified as an interesting area with benefits such as development of a biorefinary concept and easy harvest (Sankaran et al. 2010).

The fungal biomass produced during treatment of brewery wastewater could also be of interest for several applications. There is an urgent need to identify new feed resources that could increase the supply of protein and thus the sustainability in animal production (Poppi and McLennan 2010). Fungal biomass is rich in protein and fibres, and thus the cultivation of edible fungi in brewery wastewater in a controlled process may produce biomass that is useful as animal feed. Furthermore, certain fungi contain high amounts of biopolymers, such as chitin (40–45% of dry weight biomass), which make them appropriate as ingredients in biomaterial (Kumirska et al. 2010; Kuktaite et al. 2011; Dhillon et al. 2016). Another option is to use the cultivated fungal biomass in the treatment of polluted water, both due to the production of enzymes such as laccase for certain fungal species and because it has been reported that fungal biomass can replace high-cost activated carbon as a biosorbent (Parenti et al. 2013; Dhillon et al. 2016).

In this study, the main focus was on biomass production and the removal of organic carbon, phosphorus and nitrogen from brewery wastewater through submerged fungal cultivation using different species of fungi. From a longer-term perspective and considering large-scale in situ treatment, it is important to use non-harmful fungal species in order to avoid secondary health or environmental problems (Chanda et al. 2016). The fungal species tested in the present study are therefore either edible or have a long record of safe use.

## Material and methods

### Microorganisms

The fungal species used in the experiments were *Agaricus bisporus* M7215, *Pleurotus ostreatus* ATCC® 44309™ and M2140, *Lentinula edodes* M3782, *Trichoderma harzianum* CBS 226.95 and *Trametes versicolor* M9912. The species *A. bisporus*, *P. ostreatus* and *L. edodes* were selected for the experiments since they are well-known edible mushrooms. *T. versicolor* was included since it is an edible, however considered unpalatable, and fast-growing species with biotechnological and pharmaceutical application (Damle and Shukla 2010). *T. harzianum* was included since this species has a long record of large-scale use in agriculture (Vinalea et al. 2008). The strains were purchased from the American Type Culture Collection (ATCC), Mycelium BVBA, Belgium (M) and the CBS Fungal Biodiversity Centre in The Netherlands (CBS). Long-term storage of all strains was carried out at room temperature on malt agar (MA), amended with streptomycin in a concentration of 100 μg mL^−1^ in order to avoid bacterial contamination. For fungal inoculum production, all strains (with the exception of *A. bisporus*) were propagated on plates containing 20 mL potato dextrose agar (PDA) for 10 days at 27 °C. *A. bisporus* was cultivated in the same conditions for 20 days since it grew more slowly compared to the other fungi. Circular slants (diameter 15 mm) from the PDA plates were used as fungal inoculum in all the experiments.

### Brewery wastewater

Synthetic brewery wastewater (SBW), mimicking the composition of the total effluent from a brewery (Enitan et al. 2015), was prepared according to Mata et al. (2012) and used in the experiments. The composition of the SBW was 1 g L^−1^ malt extract, 0.5 g L^−1^ yeast extract, 0.15 g L^−1^ peptone, 0.86 L^−1^ maltose, 0.22 g L^−1^ (NH_4_)_2_SO_4_, 0.08 g L^−1^ NaH_2_PO_4_ and 0.14 g L^−1^ Na_2_HPO_4_. After autoclavation, the SBW was cooled and 2 mL of ethanol (96%) was added per litre. The prepared SBW had a pH of 6.6 ± 0.04. The initial values of chemical oxygen demand (COD), total nitrogen (TN), ammonium-nitrogen (NH_4_^+^-N) and phosphate-phosphorus (PO_4_^3−^-P) are presented in Table 1.

**Table 1:** Initial concentrations of the water-quality parameters chemical oxygen demand (COD), total nitrogen (TN), ammonium-nitrogen (NH_4_^+^-N) and phosphate-phosphorus (PO_4_^3−^-P) in the synthetic brewery wastewater (SBW)

Parameter	Concentration^a^
COD	5567 ± 106
Total nitrogen	111 ± 8.2
Ammonium-nitrogen	53.0 ± 6.4
Phosphate-phosphorus (PO_4_^3−^-P)	62.6 ± 4.1

All values are in mg L^−1^

^a^Mean ± SD (standard deviation)

### Experimental set-up

Experiments were performed as batch reactors in 100 mL Erlenmeyer glass flasks on a horizontal orbital shaker (VWR, Advanced 5000 shaker, Radnor, PA, USA) at 150 rpm at 27 °C. Each reactor contained 35 mL SBW. The dry weight of the inoculum (mycelium and PDA) was determined for each strain by drying slants at 60 °C until constant weight.

The first experimental set-up comprised of six treatments: (1) *A. bisporus* M7215, (2) *P. ostreatus* ATCC® 44,309™, (3) *P. ostreatus* M2140, (4) *L. edodes* M3782, (5) *T. harzianum* CBS 226.95 and (6) *T. versicolor* M9912. In the first experiment, samples were taken after 7 days of growth in order to determine fungal biomass production. Three of the included fungi that had the highest biomass production in this experiment were selected for the second experimental set-up (*P. ostreatus* M2140, *T. harzianum* CBS 226.95, *T. versicolor* M9912). During the second experimental set-up, samples were taken on days 0, 3, 6, 10 and 13 to estimate biomass production and nutrient removal over time. In these two experiments, one slant of fungal inoculum was added to each reactor.

In the third experimental set-up, a mixed culture of *P. ostreatus* M2140 and *T. harzianum* CBS 226.95 was compared to single cultures of *P. ostreatus* M2140 and *T. harzianum* CBS 226.95, respectively. Two slants of fungal inoculum were added to each reactor in the third experimental set-up, which was conducted for 8 days.

### Analysis

#### Fungal biomass production

Fungal biomass was collected by filtration through a nylon filter (mesh size 100 μm) and washed twice with an equal amount of distilled water. Before and after filtration, the filters were dried in an oven at 60 °C until constant weight in order to determine the total dry weight of the fungal biomass collected. To determine the fungal biomass produced, the dry weight of the inoculum was subtracted from the total dry weight for each fungus at the end of the experiment in the first experimental set-up.

#### Nutrient analysis

Concentrations of TN, NH_4_^+^-N, PO_4_^3−^-P and COD were determined in the SBW before and after treatment when the fungal biomass had been removed from the SBW by filtration. All SBW samples were frozen until analysis. Concentration of TN was determined with Hach Lange LCK 338 (ISO 1997) and the concentration of NH_4_^+^-N was determined with Hach Lange LCK 303 (ISO 1984). Phosphate-phosphorus was determined with Hach Lange LCK 350 (ISO 2004). In order to study the effect of the treatments on organic carbon, COD was determined using Hach Lange LCK 014 (ISO 1989).

### Statistics

In each experiment, each treatment was carried out in triplicate. Each experiment was repeated once. Mean values and standard deviations are reported. Data were analysed by analysis of variance followed by Tukey’s multiple comparison test. Differences were considered significant at *P* < 0.05 (Minitab, version 16, State College, PA, USA).

## Results

### Biomass production

**Fig. 1:**
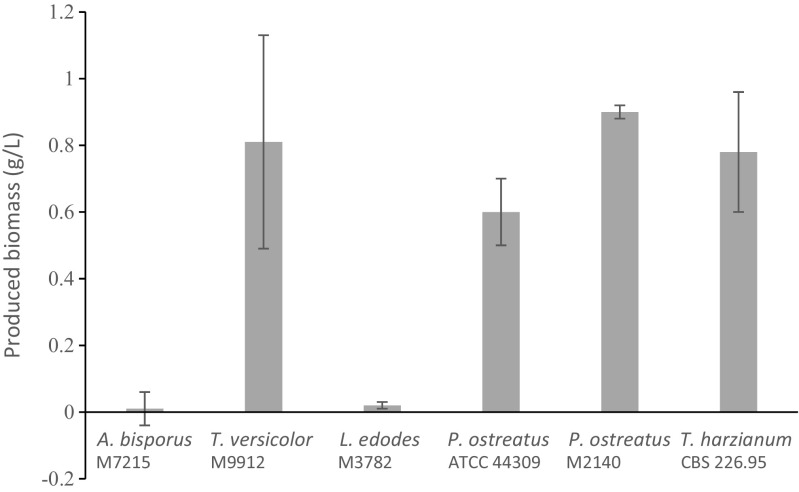
Produced biomass after 7 days of submerged growth in synthetic brewery wastewater

*P. ostreatus* M2140, *T. harzianum* CBS 226.95 and *T. versicolor* M9912 showed the highest biomass production after 7 days of growth in SBW (Fig. 1) and these were therefore selected for the second experiment. No significant differences in magnitude of biomass production were observed between these strains. *P. ostreatus* ATCC® 44,309™ showed a significantly lower biomass production after 7 days (0.60 ± 0.10 g L^−1^) compared to *P. ostreatus* M2140 (0.90 ± 0.02 g L^−1^). Very low or no biomass production was recorded for *A. bisporus* M7215 and *L. edodes* M3782 after 7 days of growth in SBW (Fig. 1).

**Fig. 2:**
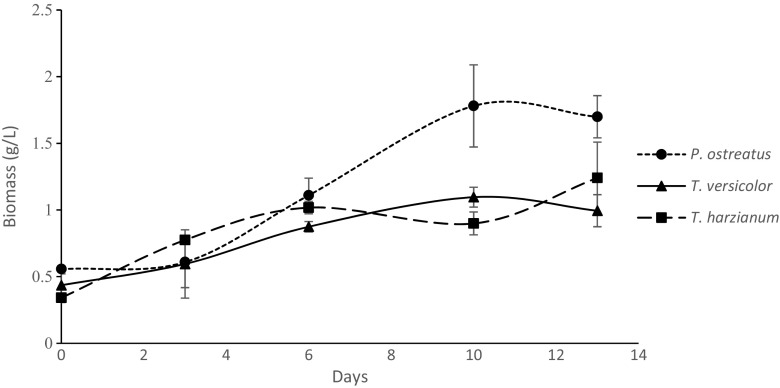
Biomass production over time for *P. ostreatus*, *T. versicolor* and *T. harzianum*, respectively during submerged growth in synthetic brewery wastewater

When biomass production over time was monitored (Fig. 2), no significant differences were observed on days 3 and 6 between the strains used. On day 10, the maximum biomass yield was noted (1.78 ± 0.31 g L^−1^) and was linked to *P. ostreatus* M2140. This biomass was significantly higher compared to the one for *T. versicolor* M9912 and *T. harzianum* CBS 226.95 on day 10. No increase in biomass was recorded between days 10 and 13 for *P. ostreatus* M2140 and *T. versicolor* M9912, while *T. harzianum* CBS 226.95 continued to increase. As a consequence of this, no significant differences were observed between *P. ostreatus* M2140 and *T. harzianum* CBS 226.95 on day 13. When a mixed culture of *P. ostreatus* M2140 and *T. harzianum* CBS 226.95 was inoculated in SBW, the biomass produced was significantly lower compared to when pure cultures were applied (Table 2). The single culture of *P. ostreatus* M2140 yielded the maximum biomass (0.96 ± 0.10 g L^−1^), which was significantly higher than the other two treatments (Table 2).

**Table 2:** Produced biomass (mg dry weight L^−1^) in synthetic brewery wastewater (SBW) after an eight-day growth period of *P. ostreatus* (PO), *T. harzianum* (TH) and PO + TH, respectively. Final concentrations (mg L^−1^) of chemical oxygen demand (COD), total nitrogen (TN), ammonium-nitrogen (NH_4_^+^-N) and phosphate-phosphorus (PO_4_^3−^-P) in the SBW are also shown

Treatment	Produced biomass	COD	TN	NH_4_^+^-N	PO_4_^3−^-P
*P. ostreatus* (PO)	955 ± 111a*	3065 ± 285a	54.2 ± 5.5a	42.8 ± 5.2a	54.2 ± 5.5a
*T. harzianum* (TH)	695 ± 44b	1221 ± 48b	53.1 ± 1.2a	17.6 ± 1.1b	53.1 ± 1.2a
PO + TH	426 ± 15c	1288 ± 104b	55.5 ± 2.2a	24.9 ± 4.3b	55.5 ± 2.2a

*Values within columns followed by different letters are significantly different (*P* < 0.05, Tukey’s test)

### Nutrient reduction

**Fig. 3:**
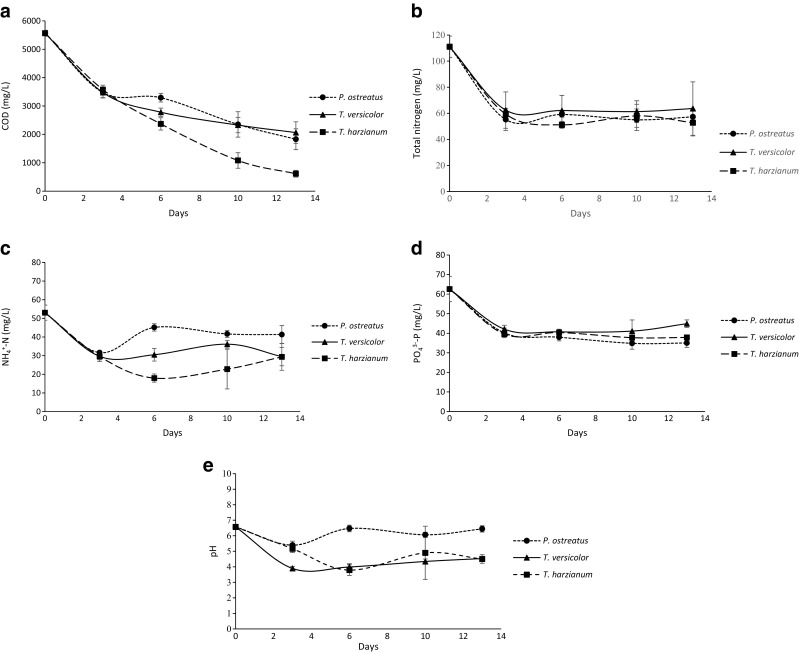
**a** Concentration of COD remaining in synthetic brewery wastewater after treatment over time by *P. ostreatus*, *T. versicolor* and *T. harzianum*, **b** concentration of total nitrogen remaining in synthetic brewery wastewater after treatment over time by *P. ostreatus*, *T. versicolor* and *T. harzianum*. **c** Concentration of NH_4_^+^-N remaining in synthetic brewery wastewater after treatment over time by *P. ostreatus, T. versicolor* and *T. harzianum*. **d** Concentration of PO_4_^3−^-P remaining in synthetic brewery wastewater after treatment over time by *P. ostreatus*, *T. versicolor* and *T. harzianum*. **e** Variation of pH in synthetic brewery wastewater after treatment over time by *P. ostreatus*, *T. versicolor* and *T. harzianum*

Concentrations of all water-quality parameters (COD, TN, NH_4_^+^-N and PO_4_^3−^-P) were significantly lower in all treatments on day 3 when compared to initial concentrations on day 0 (Fig. 3d).

All treatments resulted in a decreasing trend of COD concentrations over time (Fig. 3). On days 10 and 13, the remaining COD concentrations in the SBW were significantly lower when *T. harzianum* CBS 226.95 was applied compared to *T. versicolor* M9912 and *P. ostreatus* M2140. The maximum reduction of COD was 89.0% and was obtained on day 13 for *T. harzianum* CBS 226.95, while it was 67.1 and 62.9% for *T. versicolor* M9912 and *P. ostreatus* M2140, respectively.

For TN, there was no further decrease in concentration after day 3 and there was no significant difference between the treatments (Fig. 3b). Maximum reduction of TN was found in the *T. harzianum* CBS 226.95 treatment on day 6, corresponding to 53.9%*.* On day 13, the reduction of total nitrogen was 48.4% for *P. ostreatus* M2140, 43.0% for *T. versicolor* M9912 and 52.5% for *T. harzianum* CBS 226.95 when compared to the initial value. When NH_4_^+^-N was measured, the three treatments displayed similar concentrations on day 3; however, they differed significantly on day 6 (Fig. 3c). For *P. ostreatus* M2140, the NH_4_^+^-N concentration increased, while it was unaffected in the *T. versicolor* M9912 treatment and continued to decrease in the *T. harzianum* CBS 226.95 treatment during this period. The maximum reduction of NH_4_^+^-N was found in the *T. harzianum* CBS 226.95 treatment, 66.1%, on day 6. There was no significant difference between the treatments as regards NH_4_^+^-N concentration at day 13 (Fig. 3c).

Also, for PO_4_^3−^-P there was no further decrease in concentration after day 3 and there was no significant difference between the treatments on days 3, 6 or 10 (Fig. 3d). The maximum reduction of PO_4_^3−^-P was detected on day 10 in the *P. ostreatus* M2140 treatment (44.3%). On day 13, the reduction of PO_4_^3−^-P was 44.0% for *P. ostreatus* M2140 and 39.5% for *T. harzianum* CBS 226.95, which were significantly higher than *T. versicolor* M9912 whose reduction value was 28.3%.

When treatment of the SBW with the dual culture (*P. ostreatus* M2140 and *T. harzianum* CBS 226.95) was compared to treatment with the single cultures (*P. ostreatus* M2140 and *T. harzianum* CBS 226.95, respectively), no significant differences were observed between treatments with regard to the remaining concentrations of TN and PO_4_^3−^-P in the SBW. However, the remaining concentrations of COD and NH_4_^+^-N were significantly lower in the SBW treated with *T. harzianum* CBS 226.95, either single or dual culture, compared to the single culture with *P. ostreatus* M2140 (Table 2).

### Effect on pH

Initial pH in the SBW significantly decreased with treatments of *T. versicolor* M9912 or *T. harzianum* CBS 226.95, 4.2 ± 0.3 and 4.4 ± 0.5, respectively, while the treatment with *P. ostreatus* M2140 displayed no significant effects on initial pH, 6.4 ± 0.4. Generally, there was no significant difference between pH in the *T. versicolor* M9912 or *T. harzianum* CBS 226.95 treatments, while *P. ostreatus* M2140 had significantly higher pH throughout the study (Fig. 3e).

## Discussion

The variation in biomass production between the different fungal strains reported in the present study demonstrates the different nutritional needs and adaptation capacities of these organisms. In all three experimental set-ups, *P. ostreatus* was observed to have the highest capacity for biomass production (Fig. 1; Fig. 2; Table 2), which is in line with the notion of this species being fast growing and easily cultivated (Cohen et al. 2002).

In a previous study focusing on liquid brewery waste, higher values of biomass production for *P. ostreatus*, ranging between 3 and 20 g dry weight biomass L^−1^, have been reported (Shannon and Stevenson 1975). However, in the cited study the selected brewery wastes contained 10–30 times more COD compared to the levels in the present study and their higher biomass yield is likely to be associated to the higher content of carbon available for fungal growth. This explanation possibly also applies to *T. harzianum*, which grew well in the present study (Fig. 1; Fig. 2). Nevertheless, biomass levels were almost five times lower compared to the maximum biomass value reported by Zhang et al. (2008). In their study, *T. harzianum* had grown for 24 h in winery wastewater with COD values that were two to four times higher compared to the levels in the present study. Thus, the higher *T. harzianum* biomass obtained by Zhang et al. (2008) could also partly be explained by the higher COD values.

As mentioned above, COD concentration is an important parameter which influences the amount of biomass produced. In the brewing process, wastewater is produced in different steps and the chemical composition of the effluent from the different processes varies (Simate et al. 2011). In the present study, the COD values in the SBW corresponds well with COD levels found in combined wastewater from different brewery processes and washings (Enitan et al. 2015). It is likely that higher fungal biomass production is possible working with selected waste streams such as effluents from fermentation process and filtering. In fact, considering first-order kinetics which is commonly used to evaluate and design wastewater treatments systems, nutrient removal is a direct function of nutrient concentration, i.e. higher nutrient concentration would result in higher nutrient removal rates and higher biomass production (Henze et al. 2002). Thus, a profitable solution for breweries that would decrease the techno-economic limitations of small-scale production of fungal biomass could be to keep wastewater from different brewing processes separated and apply optimized treatment for each specific wastewater stream. Consequently, by avoiding dilution of brewery wastewater both more efficient wastewater treatment and higher fungal biomass production could be achieved.

The Swedish Agency for Marine and Water Management (HaV, Havs- och vattenmyndigheten 2016) states that 90% of the organic amount in the wastewater must be removed to adhere to current regulations for small-scale wastewater treatment systems. Despite producing a lower or similar biomass compared to the other fungal strains tested (Fig. 2), *T. harzianum* clearly displayed a superior ability over the other strains to reduce COD (Fig. 3). *T. harzianum* also seemed to be the driving force in achieving low COD levels, even in dual cultures (Table 2).Thus the capacity of *T. harzianum* to provide COD reductions of 79–89%, observed in the present study, shows a promising future application for small wastewater treatment systems, such as those at microbreweries. In the abovementioned study by Zhang et al. (2008), a COD reduction in the order of 86–91% was reported for *T. viride*, which further supports the interest of this genus in this regard. A high capacity for reduction of COD has also been demonstrated for *T. versicolor* and Singh (2006) reported 90% COD reduction by this species in anaerobically digested plant waste. In the present study, similar biomass production was observed between *T. harzianum* and *T. versicolor*; however as previously mentioned, a significantly higher reduction of COD was observed by *T. harzianum*.

**Fig. 4:**
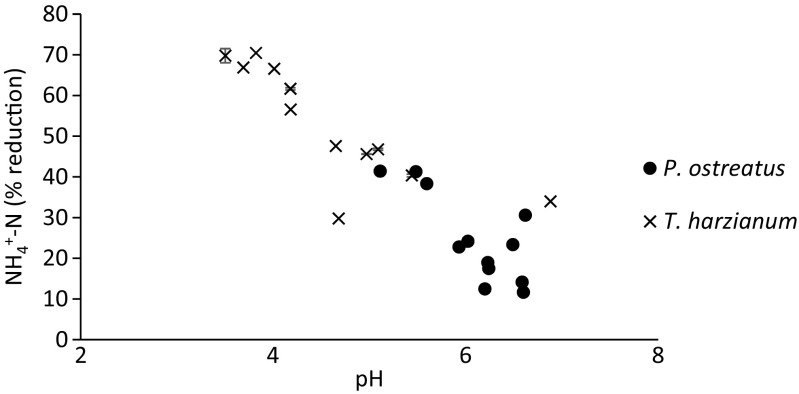
Correlation between NH_4_^+^-N reduction (%) and pH measured in synthetic brewery wastewater after treatment over time by *P. ostreatus* and *T. harzianum*, respectively

No significant difference between the treatments was found for TN (Fig. 3b). Nevertheless, *T. harzianum* was the only fungus reaching more that 50% TN reduction, which is the legal measure set by the Swedish Agency for Marine and Water Management (HaV, Havs- och vattenmyndigheten 2016) for TN removal by small-scale wastewater treatment systems. When the effect of the fungal treatments on NH_4_^+^-N was investigated, variations between the species were observed and the highest reduction was obtained by *T. harzianum* (Fig. 3c). However, all treatments exhibited increasing concentrations of NH_4_^+^-N over time, which was most likely due to mineralisation in the medium. As demonstrated in Fig. 4, the lower pH levels were associated with higher reductions in NH4^+^-N, which is in agreement with NH_4_^+^ being transported into the fungal cell as ammonia (NH_3_), leaving the hydrogen ion in the medium. Since the solubility/insolubility of many pollutants in wastewater is dependent on pH, this parameter may play an important role in the fungal treatment of different waste streams (Singh 2006). Finding optimum combinations of pH levels in the waste with the right fungal strain could hold the key to fine tuning the fungal treatment.

The different treatments showed no significant difference in their capacity to remove PO_4_^3−^-P from the SBW (Fig. 3d). Generally, the minimum COD-to-phosphorus ratio (COD:P) in biological phosphorus treatment systems is 35 g COD per g phosphorus. Also, in order to stimulate luxury uptake of wastewater phosphorus by bacteria, supplementation with short-chain organics is necessary and costly. In the present study, the COD/phosphorus ratio had a decreasing trend over time for all strains, i.e. less organic matter was needed to attain the reductions of PO_4_^3−^-P. Most striking was the decreasing COD/phosphorus ratio in the *T. harzianum* treatment from 90 on day 3 to 16 on day 13. This suggests that *T. harzianum* has a higher adaptation capacity compared to *P. ostreatus* and *T. versicolor* for an increase in carbon uptake, while keeping reduction of TN and PO_4_^3−^-P at a relatively stable level. Guest and Smith (2007) reported a low COD/phosphorus ratio of 5 when different fungi were used to treat domestic wastewater. The cited study reported reductions of PO_4_^3^-P in the range of those reported in the present study: 28.3–44%. Interestingly, a positive relationship between fungal cellular P content and increasing organic nitrogen in wastewater have been reported (Ye et al. 2015). Thus, fungi can display increasing P removal when applied to different waste streams. Consequently, in future applications, phosphorus reduction by fungi may play an important role in wastewater treatment systems, especially in systems where there is limited ability to provide the addition of appropriate organic matter in order to stimulate luxury uptake of phosphorus (Guest and Smith 2007).

According to Singh (2006), mixed fungal cultures can improve assimilation of nutrients and yield higher amounts of biomass. In the present study, a mixed culture of the main biomass-producing species, *P. ostreatus*, and the main COD-reducing species, *T. harzianum*, was tested. However, using dual fungal cultures had no advantages in the present study and in fact the dual culture had significantly lower biomass compared to the single cultures (Table 2). Furthermore, effects on water-quality parameters were not improved when using dual fungal cultures. Instead, using the single culture of *T. harzianum* in the SBW provided the most advantageous and cost-effective treatment method to achieve low levels of all the tested parameters.

In the present study very low growth was recorded for the edible species *A. bisporus* and *L. edodes*. Both species have been reported to grow under submerged conditions, and the strain *A. bisporus* MSU-2 has previously been reported to grow well in brewery waste liquor, reaching a biomass of 3–11 g L^−1^ (Shannon and Stevenson 1975; Tsivileva et al. 2010). Conversely, in the present study no biomass production was observed with *A. bisporus* (Fig. 1). Since many fungal traits such as nutrient uptake from different sources are strain specific (Singh 2006), the low biomass production might be explained by strain variation. Due to the low growth of these species, the only edible species that showed a capability for growth in the SBW was *P. ostreatus*. Neither *T. harzianum* nor *T. versicolor* are considered food sources, however both species are used on a large scale for various purposes. *T. versicolor* is a well-known laccase producer with possible biotechnological and pharmaceutical applications, and the main application of *T. harzianum* is as a biocontrol agent (Vinalea et al. 2008; Damle and Shukla 2010). Due to its high capability for reducing COD, the results from the present study suggest that *T. harzianum* is an interesting species to develop for brewery wastewater treatment. The produced biomass may then have biotechnological applications, e.g. in the production of enzymes or as ingredients in biomaterials.
